# Effect of the UK government's 2-week target on waiting times in women with breast cancer in southeast England

**DOI:** 10.1038/sj.bjc.6601149

**Published:** 2003-07-29

**Authors:** D Robinson, C M J Bell, H Møller, I Basnett

**Affiliations:** 1 Thames Cancer Registry, Guy's King's and St Thomas' School of Medicine, Capital House, 42 Weston Street, London SE1 3QD, UK; 2 Camden & Islington Health Authority, 110 Hampstead Road, London NW1 2LJ, UK

**Keywords:** breast cancer, waiting times, treatment, caseload

## Abstract

A government target of a maximum 2-week wait for women referred urgently with suspected breast cancer was introduced in April 1999. We have assessed changes in the distributions of waiting times and the proportions of cases meeting proposed targets before and after this date, using clinical audit data on 5750 women attending 19 hospitals in southeast England during the period July 1997–December 2000, who were subsequently found to have breast cancer. The proportion of cases being seen within 2 weeks of referral rose from 66.0 to 75.2%, and the median wait to first appointment fell from 13.6 to 12.3 days, following the introduction of the government target. The proportion of cases waiting 5 weeks or less between first hospital appointment and treatment fell from 83.8 to 80.3%, and median waits for treatment increased from 21.4 to 24.1 days. We also examined the effects on waiting times of various sociodemographic and care related factors. A total of 85.7% of screening cases *vs* 67.9% of symptomatic cases were seen within 2 weeks, and 95.0% of cases treated with tamoxifen received treatment within 5 weeks, as opposed to 77.6% of cases treated with surgery, 81.2% of chemotherapy cases and 52.8% of radiotherapy cases. While waiting times from GP referral to first hospital appointment have improved since the introduction of the government target, times from first appointment to treatment have increased, and consequently total waiting times have changed little.

On 1 April 1999, the UK Government introduced a ‘guaranteed’ 2-week maximum wait for assessment by a hospital consultant for women referred urgently by their GP with suspected breast cancer. Since this date, each hospital has been required to submit data to the Department of Health, and the performance of hospitals against the target is being monitored and documented on the Department's web site (www.doh.gov.uk/cancerwaits/).

However, breast clinics have been concerned for some time about increasing numbers of referrals from GPs (Patel *et al*, 2000). The predictive value of symptoms is poorly defined in general practice (Jones *et al*, 2001), and only 9–10% of referrals are subsequently found to have breast cancer (Patel *et al*, 2000; Sauven, 2001). Of these confirmed cancers, about a third are not referred urgently (Sauven, 2001), and there is concern that pressure to meet the target for urgent referrals may have resulted in longer waits for such nonurgently referred cases (Thrush *et al*, 2002). After the government target was introduced in April 1999, median waits for routine referrals increased from 2 to 3 weeks in Rotherham (Cant and Yu, 2000), and in Eastbourne they rose from 4 to 8 weeks (Khawaja and Allan, 2000).

To reduce anxiety for women with suspected cancer, all stages of the care pathway need speeding up. This was acknowledged in the NHS Cancer Plan (Department of Health, 2000), which set further targets for breast cancer patients: namely that all patients should be treated within 1 month of diagnosis by 2001, and within 2 months of urgent GP referral by 2002. These new targets are to be extended to all cancers by 2005.

Since 1996, the health authorities in and around London have been working with the Thames Cancer Registry to facilitate a regional programme of audit of breast cancer management, which aims to collect standard, comparative data and monitor quality of care (Bell and Ma, 1997), and to improve care and outcomes through an evidence-based implementation of guideline practice (Grol and Grimshaw, 1999). The project database permits the evaluation of waiting times from GP referral to first out patient appointment and to treatment in women with breast cancer, and enables exploration of the waits they experienced before and after the introduction of the government 2-week waiting time target on 1 April 1999.

## MATERIALS AND METHODS

The study area consists of the part of London north of the River Thames together with the counties of Hertfordshire and North Essex, and has a resident population of around seven million people. The 28 acute hospitals in this area have been collecting data prospectively on all new incident breast cancers since 1996. A standard clinical data set has been agreed regionally, which is similar to the current British Association of Surgical Oncology (BASO) data set. Data are collected by hospital staff and submitted annually to Thames Cancer Registry to be pooled, collated and analysed. Comparative information about case-mix and clinical quality indicators is fed back to the multidisciplinary breast teams at the hospitals (Bell *et al*, 2000). The present analysis is based on a subset of the data held on the central audit database at the Registry, which at the end of 2001 consisted of 13 234 records of female breast cancer cases first seen between January 1996 and December 2000 in one of the 28 hospitals.

Three waiting times were defined as follows: the referral wait is the interval between the date that the GP referral letter was sent and the date of first hospital attendance; the treatment wait is the interval between the date of first attendance and the date of first treatment of any kind; and the total wait is the interval between GP referral date and first treatment date. All waiting times were calculated as the number of days between relevant dates.

The NHS Cancer Plan sets targets of 2 weeks for the wait from referral to first appointment, 4 weeks from diagnosis to treatment and 2 months from referral to treatment. We have accordingly applied targets of 2 and 9 weeks for the referral wait and total wait, respectively. As date of diagnosis was not part of our data set, we have used date of first attendance as a surrogate, and applied a target of 5 weeks to the treatment wait.

Comparisons before and after the introduction of the government target were made by stratifying cases into those with GP referrals received between July 1997 and March 1999 and those received between April 1999 and December 2000, thus giving two equal 21-month intervals on either side of the introduction date of 1 April 1999.

To reduce bias due to heterogeneity between hospitals, only those that had consistently provided data over the period being examined and for which waiting times were available for the majority (>70%) of their cases were included. This helps to avoid situations where, for example, a poorly performing hospital provides data in the ‘pretarget’, but not in the ‘post-target’ period, leading to an exaggerated estimate of improvement following the introduction of the targets.

In order to maximise coverage, in cases where only one of the dates (GP referral sent and referral received) was present, the other was estimated on the basis of the finding that the median difference between these two dates in cases with both present was 1 day. Some cases were excluded because their waiting times were greater than 6 months. This was done to avoid bias due to the timing of data submission to the registry 6 months after the end of each calendar year, which resulted in a maximum possible waiting time of 6 months for cases seen at the end of a year. The proportions of cases excluded as a result of this truncation were low (0.1% for referral wait, 0.8% for treatment wait and 1.2% for total wait). The maximum observed waiting times were 281, 328 and 342 days for the referral, treatment and total waits, respectively. There were no significant differences in the proportions excluded for any of the waiting times between the periods before and after the introduction of the targets.

The final data set for analysis consisted of 5750 cases from 19 hospitals.

Waiting times were assessed in terms of both mean and median values (the distributions being highly skewed) and the proportions of cases meeting the relevant target values. Although untransformed mean values are displayed in the tables, significance tests were performed on log transformed data.

Changes in the overall distributions of the waiting times were examined by calculating the Kaplan–Meier survival curves (Kaplan and Meier, 1958), using attendance at hospital (referral wait) or commencement of treatment (treatment wait and total wait) as the outcome event. The survival curves were inverted to produce a more readily interpretable plot, showing the proportion of cases seen or treated within a given time. Differences in distributions were assessed by the log-rank test (Peto *et al*, 1977). All analyses were performed using the Stata statistical package (Statacorp, 2001).

A number of factors thought likely to affect the waiting times were studied: the patient's age at referral, deprivation index, priority (urgency of referral as assigned by the referring GP), screen detected or otherwise, the size of the clinic attended (throughput) and type of first treatment. Throughput was based on the average annual caseload over the 4 year period 1997–2000 of the hospital attended by the patient, and deprivation was assessed by assigning a quintile of the Carstairs Index (Carstairs and Morris, 1989) to each woman on the basis of her postcode of residence. Analyses were carried out by testing for differences between the proportions seen within the relevant targets at different levels of the factor concerned, using data from the complete period (July 1997–December 2000). Priority was not initially among the data items collected, and thus was only available on a small subset of 543 (9.4%) patients seen in the year 2000.

## RESULTS

Table 1

**Table 1:** Waiting time statistics for women with breast cancer, before and after 1 April 1999

**Period**	**Before (July 1997–March 1999)**	**After (April 1999–December 2000)**	**Test for difference**
Referral wait			
Proportion meeting target (%)	66.0	75.2	***
Mean (days)	13.6	12.3	***
Median (days	11	10	***
Interquartile range (days)	6–18	6–14	

Treatment wait			
Proportion meeting target (%)	83.8	80.3	**
Mean (days)	21.4	24.1	***
Median (days)	16	20	***
Interquartile range (days)	7–27	9–31	

Total wait			
Proportion meeting target (%)	89.1	88.5	(NS)
Mean (days)	35.0	36.4	*
Median (days)	29	30	**
Interquartile range (days)	17–44	18–45	

No. of cases	2712	3038	

*** *P*<0.001;

** 0.001<*P*<0.01;

* 0.01<*P*<0.025;

(NS)=*P*>0.5.

shows the changes in the three waiting times following the 1 April 1999 deadline, in terms of the mean and median waiting times and the proportion meeting the relevant target. For all three measures the referral wait showed a highly significant improvement, with decreasing waiting times and an increasing proportion meeting the 2-week target. The treatment wait showed a significant deterioration on all three measures. The changes in the total wait were small, but in the direction suggesting a worsening of total waiting times.

Figures 1

**Figure 1:**
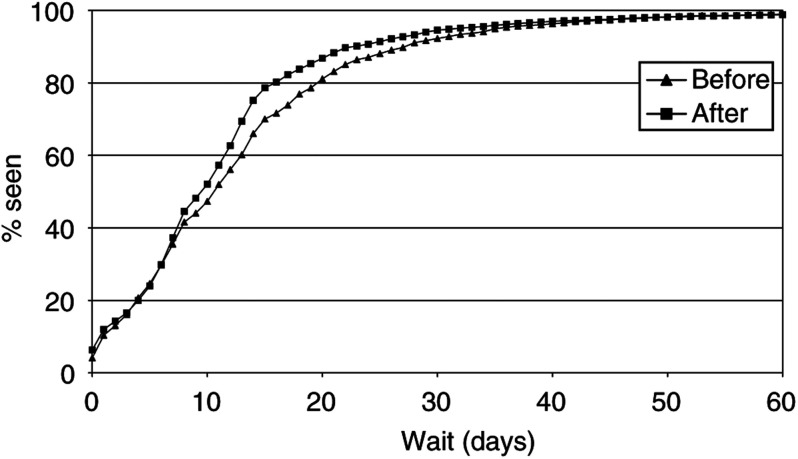
Distributions for referral wait, before and after 1 April 1999.

and 2

**Figure 2:**
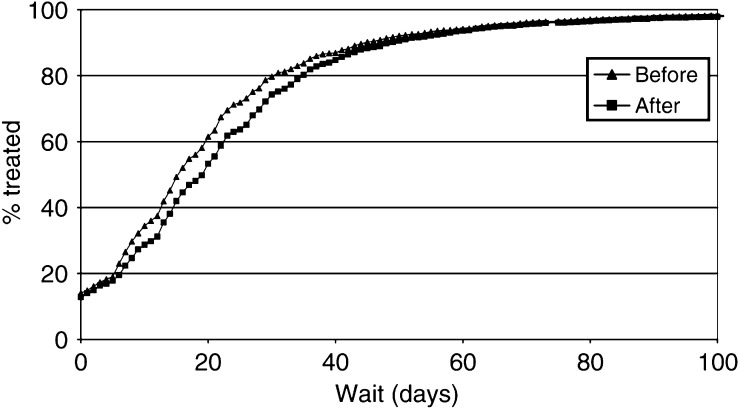
Distributions for treatment wait, before and after 1 April 1999.

show the inverse Kaplan–Meier plots for the referral wait and treatment wait, respectively, for the periods before and after 1 April 1999. There was a significant (*P*<0.001) distributional shift towards shorter waiting times for the referral wait and longer times for the treatment wait following the introduction of the target. Examination of the tails of the distributions showed that there was no suggestion of an increase in the number of cases waiting for very long periods. No significant change in the distribution of total wait values was seen (not shown).

Considerable variation was seen between hospitals. In eight of the 19 hospitals, the proportion meeting the target for the referral wait increased by more than 10 percent following the April 1999 deadline, but the proportion meeting the corresponding target for the treatment wait deteriorated significantly in half of these. Only two hospitals showed a substantial improvement (>5% increase in the proportion meeting the target) in both waiting times, and one hospital displayed a substantial deterioration in both.

Table 2

**Table 2:** Proportions (%) meeting relevant targets by various factors (July 1997–December 2000)

**Factor**	**Referral wait**	**Treatment wait**
Age (years) (*n*=5592)		
<50	64.2	77.3
50–64	76.5	80.5
65+	70.0***	85.6***

Carstairs quintile (*n*=3458)		
1	70.7	79.8
2	65.7	80.4
3	66.8	82.8
4	67.1	82.7
5	67.5(NS)	82.7(NS)

Trust throughput (*n*=5750)		
−150	69.7	80.6
>150	72.1*	83.3**

Priority (*n*=543)		
Urgent	77.2	
Nonurgent	41.2***	

Mode of presentation (*n*=5450)		
Screening	85.7	
Symptomatic	67.9	
Incidental	77.7***	

Type of treatment (*n*=5701)		
Surgery		77.6
Chemotherapy		81.2
Radiotherapy		52.8
Tamoxifen		95.0***

Test for heterogeneity:

*** *P*<0.001;

** 0.001<*P*<0.01;

* 0.01<*P*<0.025;

(NS)=*P*>0.5.

a Two week target for referral wait; 5 week target for treatment wait.

shows the proportions meeting the relevant targets in relation to the various explanatory factors studied. By age, the highest proportions meeting the target were seen in the 50–64 years age group for the referral wait, and the 65+ years age group for the treatment wait. Although for the referral wait a higher proportion of Carstairs group 1 cases (the most affluent) were seen within 2 weeks when compared to the other groups, the overall test for differences between the Carstairs quintile groups was nonsignificant (*P*=0.7). For both referral wait and treatment wait, a significantly higher proportion of women attending high throughput hospitals met the targets. A significantly greater proportion of urgent referrals were seen on target for the referral wait.

There were large differences for the referral wait in relation to mode of presentation, with a larger proportion of women with screen-detected cancers meeting the target. Likewise, for the treatment wait large differences were seen for different types of treatment. Approximately 95% of those receiving tamoxifen or endocrine treatment were treated within 5 weeks, as opposed to only 53% of those receiving radiotherapy as their first treatment.

**Figure 3:**
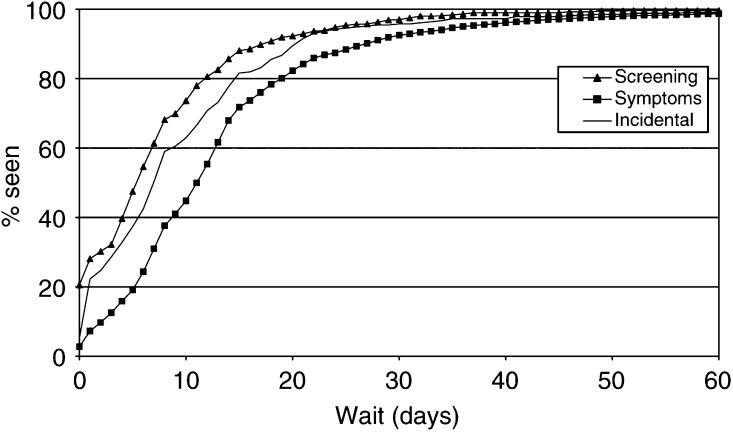
Referral wait distributions by mode of presentation (July 1997–December 2000).

The inverse Kaplan–Meier plots for referral wait in relation to mode of presentation and treatment wait in relation to treatment type are shown in Figures 3

and 4

**Figure 4:**
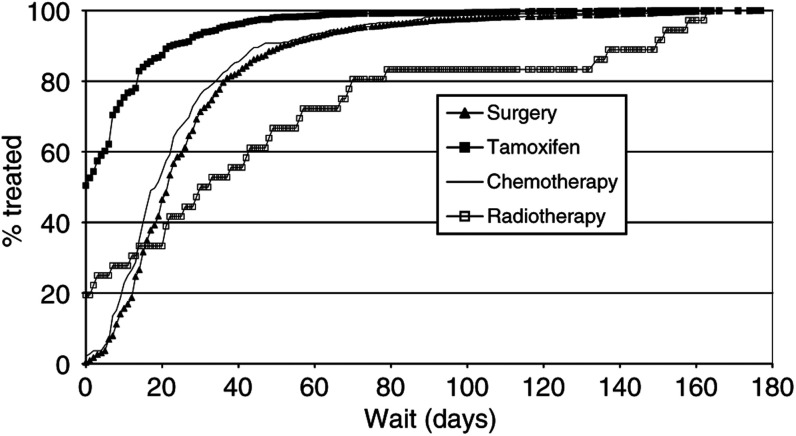
Treatment wait distributions by type of treatment (July 1997–December 2000).

, respectively. The distributional differences are striking, and log-rank tests were highly significant.

## DISCUSSION

There has been a significant improvement in waiting times from GP referral to first hospital appointment in women with breast cancer following the introduction of the ‘guaranteed’ 2-week maximum wait in April of 1999. However, there has been a corresponding deterioration in the waiting times between first hospital appointment and treatment, with the result that total waiting times remain relatively unchanged.

There was no evidence that any group (e.g. nonurgent cases) was particularly disadvantaged by the introduction of the 2-week wait target. When the distributions of referral waiting times before and after the 1 April 1999 deadline were compared, there was no suggestion of the crossover that would be expected if a substantial number of cases were being made to wait longer. There was a shift towards shorter waits in the later period across the whole range of waiting times.

If the aim of urgent referral is to detect cancers quickly, as well as assuage anxiety, then it may be that priority should be assigned by consultants. We have found evidence to suggest that consultants are better than GPs at correctly prioritising the patients with cancer, as suggested previously by Sauven (2001). In our data set, 340 cases had data on priority assigned by both consultant and GP in the post-target period. For the vast majority of these (314, 92%), the GP and consultant concurred about urgency. However, 21 women (6%) referred as nonurgent by the GP were designated as urgent by the consultant. This small group of women had a mean wait of 20.9 days (compared with 12.3 days overall) and a median wait of 14 days compared with 10 overall. Three of these women waited over 1 month, and one several months, for a first appointment.

Government targets relating to the second part of the patient pathway, from first hospital appointment to treatment, were not introduced until 2000. Our data showed that the median wait for first treatment was 4 days longer, and the proportion treated within 5 weeks of first hospital appointment was 3.5% less, after the introduction of the 2-week target for the referral wait in 1999. One explanation for this might be that the pressure to shorten the referral wait, without additional resources being available in hospitals to deliver an improved service, has led to delays in treatment. The effects we have observed reflect the risks inherent in a policy of targeting one element of the patient pathway in isolation. The new targets, covering the whole of the patient pathway from GP referral to treatment, should be better able to ensure an improved patient experience.

The proportion meeting the target is generally higher for the treatment wait than for the referral wait, partly because the diagnosis is established by the beginning of the second interval. The tumours have by this time been confirmed as malignant, and are therefore all considered urgent. There may also be more flexibility within the longer second target period to compensate for small hold-ups at various points along the pathway.

The observed age effect on the referral wait, with waiting times significantly shorter in the age group 50–64 years, may be due to screening–screen-detected patients had shorter waits for outpatient appointments. Likewise, the increased proportion of cases in the oldest age group meeting the target for the treatment wait may be due to treatment modality–older patients are more likely to receive tamoxifen as a first treatment, and there was little or no delay in starting this.

Treatment wait was significantly influenced by only two factors–it was shorter in high throughput hospitals, and highly dependent on the type of first treatment. Although the majority of the breast cancer cases in our study (93%) received surgery or tamoxifen as their first treatment, the small group of women who received radiotherapy as their first treatment experienced long delays, with little more than half being treated within the target time of 5 weeks. Moreover, the situation deteriorated following the introduction of the government targets, with median waiting times for radiotherapy increasing from 29 to 43 days.

There was also a greater proportion of treatment waiting times for radiotherapy (14%) that had been excluded as being longer than 6 months, compared to other treatment modes (1%). Had these cases been left in the analysis, then the disparity would have been even greater.

A combined analysis of data from 21 breast cancer studies (Huang *et al*, 2003) has shown that delay in the initiation of radiotherapy is associated with an increase in the 5-year local recurrence rate. Although additional resources have been allocated within the NHS for radiotherapy, there is still a shortage of trained radiographers and a recent survey by the Royal College of Radiologists has shown that treatment delays are increasing (Browne, 2002).

On the other hand, data from the most recent year of our audit (2001) show considerable improvements for radiotherapy waiting times, with a median delay of 18 days and 75% of cases being treated within 5 weeks of their first hospital appointment.

Tamoxifen was the first form of treatment in 26% of cases, surgery in 68%, chemotherapy in 6% and radiotherapy in less than 1%. These proportions were dependent on age: in women aged <65 years at referral, 79% had surgery and 12% tamoxifen as first treatment, whereas in those aged 65 or over the corresponding proportions were 51 and 47%, respectively. Of the ‘tamoxifen first’ cases, 47% had subsequent surgery (with a median subsequent wait of 27 days), 11% had chemotherapy (median wait 56 days) and 20% radiotherapy (median wait 120 days).

In our data, there were no significant changes in the proportions receiving different kinds of treatment following the government mandate, suggesting that the observed overall increases in treatment waits are not due to changes in treatment policy. Indeed, increases in median waiting times were observed for all treatment modalities.

Our results give a clear message about the need to explore in detail the statistics used to represent performance on waiting times. While the simple summary measures employed produce broadly consistent results, the ‘proportion meeting a target’ is a poor statistic to represent the experience of the group of patients as a whole. Median waits can also be insensitive to changes in the tails of the distributions. The Kaplan–Meier plots are both informative for the population as a whole and sensitive enough to show differences in the experience of subgroups within the population.

Not all 28 hospitals in our area were represented in the study. Only those women attending the 19 hospitals that consistently supplied data of sufficiently high quality for the comparative audit were included. If there were any correlation between organisation of the audit and organisation of the patient pathway in hospitals, then this analysis may be biased in favour of overestimating the improvement in waiting times as a result of the government's strategy. Indeed, analysis of the data from the excluded hospitals tends to corroborate this, with smaller increases or greater decreases in the proportions meeting the relevant targets.

By definition, all the women in our analysis had a treatment date of some kind recorded. However, to be classified as the mode of first treatment, any particular treatment type would need to have a nonmissing date for the start of that treatment. In general, these dates were well recorded with the poorest being for radiotherapy, where only 63% of the cases known to have received radiotherapy had a corresponding date present. It is therefore possible that some of these cases may have been misclassified, but as most would have undergone previous surgery any resulting bias is likely to be small.

Our study describes the experience of women who have been diagnosed with breast cancer, as opposed to those suspected of having breast cancer. As such, we felt it was appropriate to include screen-detected cases. Reanalysis after excluding screening cases does not materially affect any of our findings. Some 20% of the lesions in our screen-detected cases were *in situ* carcinomas, compared with 5% in nonscreening cases. It is perhaps ironic therefore that the screening cases should enjoy more rapid access to services than clinical cases. Our study adds weight to the fears that because many breast cancers present among symptomatic ‘nonurgent’ cases, the waits after screening give a falsely optimistic picture.

The targets in the NHS Cancer Plan to reduce the wait from referral to first appointment look achievable, but a guaranteed minimum wait for 100% of patients is almost impossible (Jones, 2001). Even with substantial overprovision of appointment slots, random variations in demand will exceed supply at times. In contrast, a 95% or even 99% target is practicable provided the number of GP referrals does not increase as waits decrease. Waits for treatment are more amenable to planning (Thomas and Burnet, 2001), as the number of cancer patients per annum (the incidence rate) is fairly stable. Reducing waits for treatment is also more valuable to patients in terms of survival outcomes (Richards *et al*, 1999) and tumour control (Wyatt *et al*, 2003).

Our data show that waiting times can be shortened, and the additional resources made available since 2000 within the NHS Cancer Plan, along with the introduction of targets for waiting times from GP referral to treatment, should assist hospitals in speeding up the whole of the patient pathway.

## References

[bib1] Bell CMJ, Ma M, Campbell S, Basnett I, Pollock A, Taylor I (2000) Methodological issues in the use of guidelines and audit to improve clinical effectiveness in breast cancer in one United Kingdom health region. Eur J Surg Oncol 26: 130–136 10744929 10.1053/ejso.1999.0755

[bib2] Bell J, Ma M (eds) (1997) Prospective Audit of Breast Cancer: the First Year. London: Thames Cancer Registry

[bib3] Browne A (2002) Deadly rise in wait for cancer care. Guardian, Sunday March 3

[bib4] Cant PJ, Yu DS (2000) Impact of the ‘2 week wait’ directive for suspected cancer on service provision in a symptomatic breast clinic. Br J Surg 87: 1082–1086 10931055 10.1046/j.1365-2168.2000.01551.x

[bib5] Carstairs V, Morris R (1989) Deprivation and mortality: an alternative to social class? Comm Med 11: 210–219 10.1093/oxfordjournals.pubmed.a042469 2605887

[bib6] Department of Health (2000) The NHS Cancer Plan. London: Stationery Office

[bib7] Grol R, Grimshaw J (1999) Evidence-based implementation of evidence-based medicine. Jt Comm J Qual Improv 25: 503–513 10522231 10.1016/s1070-3241(16)30464-3

[bib8] Huang J, Barbera L, Brouwers M, Browman G, Mackillop WJ (2003) Does delay in starting treatment affect the outcomes of radiotherapy? A systematic review. J Clin Oncol 21: 555–563 12560449 10.1200/JCO.2003.04.171

[bib9] Jones R (2001) Waiting times. Quick quick slow. Health Serv J 111: 20–23 11695061

[bib10] Jones R, Rubin G, Hungin P (2001) Is the two week rule for cancer referrals working? BMJ 322: 1555–1556 11431280 10.1136/bmj.322.7302.1555 PMC1120606

[bib11] Kaplan EL, Meier P (1958) Nonparametric estimation from incomplete observations. J Am Statist Assoc 53: 457–481

[bib12] Khawaja AR, Allan SM (2000) Has the breast cancer ‘two week wait’ guarantee for assessment made any difference? Eur J Surg Oncol 26: 536–539 11034802 10.1053/ejso.2000.0942

[bib13] Patel RS, Smith DC, Reid I (2000) One stop breast clinics–victims of their own success? A prospective audit of referrals to a specialist breast clinic. Eur J Surg Oncol 26: 452–454 11016464 10.1053/ejso.1999.0920

[bib14] Peto R, Pike MC, Armitage P, Breslow NE, Cox DR, Howard SV, Mantel N, McPherson K, Peto J, Smith PG (1977) Design and analysis of randomized clinical trials requiring prolonged observation of each patient. II: analysis and examples. Br J Cancer 35: 1–39 831755 10.1038/bjc.1977.1 PMC2025310

[bib15] Richards MA, Westcombe AM, Love SB, Littlejohns P, Ramirez AJ (1999) Influence of delay on survival in patients with breast cancer: a systematic review. Lancet 353: 1119–1126 10209974 10.1016/s0140-6736(99)02143-1

[bib16] Sauven P (2001) Specialists, not GPs, may be best qualified to assess urgency. BMJ 323: 864–865 11683156

[bib17] Statacorp (2001) Stata Statistical Software: Release 7.0. College Station: Stata Corporation

[bib18] Thomas S, Burnet N (2001) Reducing waiting times from diagnosis to treatment might be more effective. BMJ 323: 864 PMC1121397 11683154

[bib19] Thrush S, Sayer G, Scott-Coombes D, Roberts JV (2002) Grading referrals to specialist breast unit may be ineffective. BMJ 324: 1279 10.1136/bmj.324.7348.1279/a PMC1123230 12028993

[bib20] Wyatt RM, Beddoe AH, Dale RG (2003) The effects of delays in radiotherapy treatment on tumour control. Phys Med Biol 48: 139–155 12587901 10.1088/0031-9155/48/2/301

